# Short‐Term Dietary Intervention Alters Physiological Profiles Relevant to Ageing

**DOI:** 10.1111/acel.70507

**Published:** 2026-04-27

**Authors:** Caitlin J. Andrews, Rosilene V. Ribeiro, Alison Gosby, David G. Le Couteur, David Raubenheimer, Jian Tan, Stephen J. Simpson, Alistair M. Senior

**Affiliations:** ^1^ School of Life and Environmental Sciences University of Sydney Sydney New South Wales Australia; ^2^ Charles Perkins Centre University of Sydney Sydney New South Wales Australia; ^3^ ANZAC Research Institute, Concord RG Hospital and Faculty of Medicine and Health, School of Medical Sciences University of Sydney Concord New South Wales Australia; ^4^ Chronic Disease Theme, School of Medical Sciences, Faculty of Medicine and Health University of Sydney Sydney New South Wales Australia

## Abstract

Ageing is a complex process influenced by modifiable factors such as diet, which may accelerate or decelerate physiological decline. While chronological age increases uniformly, biological ageing varies between individuals, reflecting differences in health status and the resilience of biological systems. The Klemera‐Doubal Method (KDM), a composite biomarker‐based index often used as an estimate of biological age, has been associated with morbidity and mortality in large cohorts. This study examined whether dietary manipulation of protein source and macronutrient composition affects KDM estimates in older adults. We analysed data from the Nutrition for Healthy Living study, a 2 × 2 factorial dietary intervention trial involving 104 participants aged 65–75 years. Participants were randomised to one of four diets: omnivorous/high‐fat (OHF), omnivorous/high‐carbohydrate (OHC), semi‐vegetarian/high‐fat (VHF) or semi‐vegetarian/high‐carbohydrate (VHC). KDM‐derived δAge (the difference between KDM‐ and chronological‐age) was calculated before and after a 4‐week intervention. The OHF group, most like participants' baseline diets, showed no meaningful change in δAge. Compared to OHF, participants in the OHC group showed a significant reduction in δAge. The VHF and VHC groups showed similar reductions in δAge, relative to OHF, though not all reached statistical significance. KDM‐derived δAge appears responsive to dietary change within 4 weeks and may offer a useful proxy for evaluating shifts in physiological status. Caution is warranted in interpreting such changes as evidence of biological age reversal as observed shifts may reflect acute physiological responsiveness to dietary inputs rather than altered ageing trajectories. Longer‐term treatment would be needed to assess changes in age‐related disease risks.

## Graphical abstract

A 4‐week controlled dietary intervention in older adults reduced KDM‐derived δAge, especially with high‐carbohydrate and semi‐vegetarian diets, indicating rapid shifts in age‐related physiological profiles. These changes likely reflect coordinated, system‐wide physiological adaptations to dietary intake, with potential implications for longer‐term biological ageing trajectories.

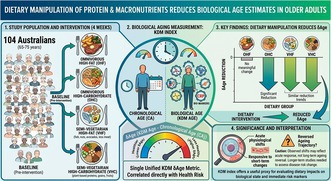

## 1 Introduction

Ageing is a nearly universal biological process, characterised by complex multifactorial and interconnected molecular, cellular, and physiological changes (López‐Otín et al. 2023, 2013). Regardless of its underlying causes, ageing is typically associated with a decline in health and an increased risk of morbidity and mortality. However, the rate at which individuals experience age‐related decline, relative to their chronological age (CA), can vary widely and is influenced by various factors. Among these, diet stands out as a key modifiable determinant that may influence physiological health and modulate age‐associated biomarker profiles (Le Couteur et al. 2024). The impact of dietary protein sources and macronutrient composition on ageing remains a topic of ongoing debate. Evidence suggests that both the quantity and source of dietary protein, whether animal‐based or plant‐based, may differentially influence biomarkers linked to inflammation, oxidative stress and other ‘hallmarks’ of ageing (Song et al. 2016). For instance, animal‐based proteins, such as meat and dairy, are often linked to elevated levels of pro‐inflammatory and oxidative biomarkers (Beasley et al. 2014; Vitale et al. 2012; Franceschi et al. 2005), known accelerators of the ageing process (Guest 2019; Tao et al. 2023; Wang, Hou, et al. 2023). In contrast, plant‐based proteins, found in foods like legumes and whole grains, are typically associated with more favourable biomarker profiles (Wang, Li, et al. 2023; Stoodley et al. 2023; Ardisson Korat et al. 2024; Acosta‐Navarro et al. 2019; Craddock et al. 2019). Distinct age‐related health outcomes have been observed between vegetarian and omnivorous dietary patterns, which differ in their relative proportions of animal‐ and plant‐based protein (Ortolá et al. 2020). These dietary patterns have been linked to differences in metabolic health, cardiovascular health, cognitive performance and overall longevity (Petermann‐Rocha et al. 2021; Appleby and Key 2016; Herpich et al. 2022).

In addition to protein source, interactions between macronutrients may also influence ageing trajectories (Mirzaei et al. 2016; Okburan and Gezer 2021). For instance, high‐carbohydrate, low‐protein diets have been shown to extend lifespan in animal models and improve metabolic health in humans (Le Couteur et al. 2024, 2016; Okburan and Gezer 2021; Kitada et al. 2019; Lagiou et al. 2007; Solon‐Biet et al. 2015). Meanwhile, high‐fat diets have produced both beneficial and adverse health outcomes, depending on context and composition (Halade et al. 2010; Guasch‐Ferré et al. 2015; Ramón et al. 2018; Lands 2005; Ho et al. 2020; Jyväkorpi et al. 2020; Päivärinta et al. 2020; Bernstein et al. 2010). These findings raise questions about whether the observed changes in health‐related biomarkers under different dietary patterns are attributable to fat content rather than protein per se (Campbell 2017; Fung et al. 2010; Zong et al. 2018), or whether specific types of carbohydrates in plant‐based diets are responsible for observed benefits (Fabek et al. 2021; De Natale et al. 2009; McMacken and Shah 2017; Kadyan et al. 2022; Warman et al. 2022; Kahleova et al. 2018).

Because CA alone cannot capture variation in physiological decline due to genetic, lifestyle or environmental influences, researchers have developed alternative measures of biological age that integrate diverse biomarkers associated with ageing. These composite indices aim to reflect the functional state of the body relative to age norms and have been designed with the intention to quantify the cumulative degradation of system integrity driven by underlying molecular and cellular processes.

One such method, the Klemera‐Doubal Method (KDM) (Wei et al. 2022; Mak et al. 2023; Parker et al. 2020) estimates age‐relative physiological status by integrating a suite of blood and clinical biomarkers known to vary systematically with CA (Cao et al. 2021) at the population level. Rather than focusing on specific disease outcomes, the KDM generates a composite indicator (KDMAge) of multisystem physiological functioning that typically varies with age and predicts age‐related outcomes but may also respond to non‐ageing‐related influences (Cao et al. 2021; Levine 2013; Kwon and Belsky 2021).

We refer to the difference between CA and KDMAge as δAge, representing the extent to which an individual's biomarker profile aligns with or diverges from age‐typical expectations. A smaller δAge value indicates that an individual's physiological status is more closely aligned with what is typical for their age and sex, while a larger positive or negative δAge reflects deviation from this norm. A positive δAge (i.e., KDMAge > CA) indicates a deviation toward an older physiological profile than expected for one's CA and sex, and in previous studies has been generally associated with reduced life expectancy and increased risk of age‐related diseases in longitudinal cohorts (Sluiskes et al. 2024). Conversely, a negative δAge value (KDMAge < CA) suggests a more favourable state of physiological resilience relative to age norms. Notably, estimates of δAge derived from the KDM algorithm have been shown to outperform CA when predicting age‐related morbidity and mortality across diverse populations (Levine 2013).

Several cohort studies have demonstrated associations between diet and measures of age‐related status (Thomas et al. 2023; Senior et al. 2022). For instance, one study using the epigenetic clock PhenoAge, based on DNA methylation markers, found that dietary components such as total fat, added sugar and starchy vegetables were associated with increased epigenetic ageing estimates, while nuts, poultry and discretionary oils were linked to healthier values (Biemans et al. 2024). Another study linked pro‐inflammatory diets with increased KDM and PhenoAge estimates (Xie et al. 2023). More recently, clinical trials have begun to test whether dietary interventions can causally influence these measures. A 2‐year calorie‐restriction trial reduced some, but not all, epigenetic estimates of ageing (Waziry et al. 2023). Similarly, Dwaraka et al. (2024) found that an 8‐week plant‐based diet reduced measures of epigenetic age acceleration in twin pairs relative to a healthy omnivorous diet, suggesting that dietary protein source may influence short‐term shifts in biological age estimates.

It is worth noting that the interpretation of such short‐term changes remains uncertain. A recent longitudinal study, for example, found that pregnancy temporarily increased epigenetic age by up to 2 years, followed by a reversal of up to 8 years postpartum (Pham et al. 2024). This suggests that biological age estimates are responsive to transient physiological states, and that short‐term deviations may not necessarily reflect long‐term changes in ageing processes.

Here, we sought to test whether dietary manipulation could influence KDM‐derived δAge, a proxy for age‐related physiological status. To address this, we applied the KDM algorithm to data from the Nutrition for Healthy Living (NHL) study (Ribeiro et al. 2019), a 2 × 2 factorial dietary trial involving 104 older‐adults (aged 65–75 years). Participants were randomised to one of four diets: omnivorous high‐fat (OHF), omnivorous high‐carbohydrate (OHC), semi‐vegetarian high‐fat (VHF) or semi‐vegetarian high‐carbohydrate (VHC). Biomarkers were measured at baseline and after a 4‐week intervention, during which all foods were delivered to participants.

To our knowledge, this is the first study to examine the effects of a fully controlled dietary intervention on KDMAge in an older population. While the intervention period was relatively short, this design enables us to test whether contrasting dietary patterns can induce measurable changes in age‐associated biomarker profiles. It offers an opportunity to evaluate the responsiveness of δAge to diet, and to critically consider whether observed shifts reflect acute physiological flexibility or could, if sustained, signal the beginnings of altered ageing trajectories.

## 2 Methods

### 2.1 NHL

The NHL trial was fully described in Ribeiro et al. (2019). Briefly, NHL involved older adults aged between 65 and 75 years with a body mass index (BMI) ranging from 20 to 35 kg/m^2^. Recruited individuals were non‐smokers and non‐vegetarians, with no serious health complications (e.g., type‐2 diabetes mellitus, cancers, renal or liver disease) or food allergies and/or intolerances. Participants were subjected to a dietary intervention involving four diets, varying in protein source and macronutrient ratio. The study was designed as a single‐blinded, randomised clinical trial with a 2 × 2 factorial dietary design. Diets were either omnivorous or semi‐vegetarian both with 14% of energy from protein and either high fat/low carb (37%–41% of energy from fat and 41%–43% from carbs) or the reverse (28%–29% fat; 53% carbohydrate). Each participant was thus randomly assigned to one of the four following diets: omnivorous high‐fat (OHF), omnivorous high‐carb (OHC), semi‐vegetarian high‐fat (VHF) or semi‐vegetarian high‐carb (VHC). Participants were provided with all meals for a duration of 4 weeks via a food delivery program. Participants were allowed to consume from the set menu ad libitum and were deliberately provided with an excess of food allowing them to eat freely (i.e., without an upper limit). Various health assessments were conducted before and after the intervention, including cardio‐metabolic, body composition and physical performance. Based on weighed food records, participants were considered highly compliant, with the diets achieving the target changes in macronutrient intake, and protein source (Ribeiro et al. 2024).

### 2.2 KDMAge Estimation

While the concept of biological age remains debated, and multiple algorithms exist, we use KDMAge as a practical, biomarker‐based proxy to evaluate group‐level shifts in age‐relative physiological status in an older cohort. The KDMAge algorithm, originally proposed by Levine (Levine 2013), is based on a series of single‐parameter regressions of individual biomarkers against CA within a reference or ‘training’ population. The regressions fitted from the training population are then used to estimate KDMAge (in years) using corresponding biomarker values from individuals within a separate focal population of interest. Following Levine (Levine 2013) and Kwon and Belsky (Kwon and Belsky 2021), we utilised data from the National Health and Nutrition Examination Survey (NHANES) as our reference population [available from the US Centers for Disease Control and Prevention: https://wwwn.cdc.gov/nchs/nhanes/]. NHANES was conducted by the National Centre for Health Statistics in the United States and combines interview and physical examination data to provide a nationally representative picture of the general health and nutritional status of the population. We utilised data, including demographic, biomarker, functional performance, and mortality data, from the NHANES III (1988–1994) and NHANES IV (1999–2018) datasets. NHANES III data were downloaded from https://doi.org/10.6084/m9.figshare.21743372.v6. NHANES IV data were downloaded from https://wwwn.cdc.gov/nchs/nhanes using the RNAHNES R package (Susmann 2016). All data were analysed, and calculations made in R (v4.3.3). KDM algorithms were implemented using the ‘kdm_calc’ function from the BioAge package R package (Kwon and Belsky 2021).

The biomarkers available for the NHL study differed slightly from those previously described by Levine (Levine 2013). We therefore calculated KDMAge using two different sets of biomarkers (Table 1). The first method used five of the nine biomarkers originally used by Levine (Levine 2013) that were available for NHL, as well as two markers that were logical substitutes for those used by Levine (Levine 2013); we refer to the estimate of KDMAge based on this substituted set of biomarkers as ‘KDM‐S’. Secondly, we used the same overall approach followed by Levine (Levine 2013) in deriving their biomarker set (Levine 2013) to derive a completely new measure of KDMAge. This second method was based on the union of biomarkers available in all NHANES III, NHANES IV and NHL; this estimate, based on a maximal set of biomarkers, has been referred to as ‘KDM‐M’.

**TABLE 1:** Biomarker sets used for each algorithm variant used to estimate KDMAge.

Biomarkers	KDMAge algorithm		
	KDM‐L	KDM‐S	KDM‐M
Forced expiratory volume (mL)	●		
Glycohemoglobin (%)	●		
Blood urea nitrogen (mg/dL)	●		
C‐reactive protein (mg/L)	●	●	●
Albumin (g/L)	●	●	●
Total cholesterol (mmol/L)	●	●	●
Systolic blood pressure (mmHg)	●	●	●
High‐density lipoprotein (mmol/L)			●
Diastolic blood pressure (mmHg)			●
Insulin (pmol/L)			●
Alkaline phosphatase (U/L)	●	●	●
Aspartate aminotransferase/Alanine aminotransferase ratio			●
Total bilirubin (umol/L)			●
Triglycerides (mmol/L)			●
Serum glucose (mmol)		●	●
Urine creatinine (mmol/L)		●	●
Serum creatinine (mg/dL)	●		
Waist circumference (cm)			●
BMI			●
Total number of biomarkers	9	7	15

When defining KDM‐M, if multiple biomarkers for the same system were strongly correlated, only one was included. This follows the advice of Klemera and Doubal (Klemera and Doubal 2006) who emphasise the use of functionally uncorrelated markers to enhance the precision of KDMAge estimates by minimising the variance. All KDMAge algorithms were trained sex‐specifically using biomarker data from non‐pregnant participants aged 30–75. In training the algorithms, biomarker values that were more than 5 standard deviations from the sex‐specific mean for that biomarker were considered as outliers and excluded, following Kwon and Belsky (2021). Referring to Kwon and Belsky (2021), biomarkers with non‐normal distributions (AST/ALT ratio, C‐reactive protein, urinary creatinine, serum glucose, serum insulin, serum total bilirubin and serum triglycerides) were log transformed to better meet the assumption of normality of residuals. For consistency across datasets urine creatinine and serum glucose values were converted using the following conversion factors: 1 mg/dL = 88.4 umol/L for creatinine, 1 mg/dL = 10 mg/L for c‐reactive protein and 1 mg/dL = 55.5 umol/L for glucose.

Prior to testing for effects of diet on KDM‐derived δAge in the NHL study, a series of analyses were first conducted using NHANES III and IV to compare KDM‐S and KDM‐M, with KDMAge estimated using exactly the same set of biomarkers originally used by Levine (2013), referred to as ‘KDM‐L’. KDM‐L was calculated using the BioAge package, based on the original KDM algorithm in Levine (2013). In making these comparative analyses, we trained the algorithms on NHANES III data and tested their predictive power for mortality in NHANES IV.

Finally, we calculated KDM‐S and KDM‐M for individuals in the NHL dataset using NHANES IV as the reference population. KDM‐S and KDM‐S‐derived δAge was calculated for 104 NHL participants with complete sets of biomarker data (male *n* = 34; female *n* = 70). Due to missing biomarkers, one participant (male; OHC) was dropped from the KDM‐M analyses.

### 2.3 Statistical Analysis

#### 2.3.1 Analysis of NHANES III and IV

We first examined correlations between our different KDMAge measures, as well as with CA within the different NHANES cohorts. Following Levine's analysis (Levine 2013) we also examined the associations between KDM‐derived δAge and mortality in a sex specific manner in both the NHANES III reference population and the NHANES IV test populations to determine the predictive power of each measure in relation to mortality. Mortality Hazard Ratios (MHR) estimated from Cox proportional hazard regressions as well as 95% confidence intervals (CIs) were calculated for each KDMAge measure. To help clarify the potential implications of the KDM measures on mortality, δAge values were standardised sex‐specifically around a mean of 0 and standard deviation of 1. Therefore, an MHR greater than 1.0 indicates that δAge is predictive of increased mortality risk.

#### 2.3.2 Analysis of NHL

Multiple linear regression models were used to analyse the effect of the dietary interventions on KDM‐S‐ and KDM‐M‐ derived δAge. Effects were estimated for all diets relative to the OHF diet group; the OHF diet was chosen to serve as the reference diet as it most closely resembled the participants' standard/baseline (i.e., pre‐trial) diet in terms of nutritional composition (Ribeiro et al. 2019). Linear models were implemented using the *lm* function from the *R stats* package. All models included baseline KDM‐derived δAge and diet as predictors, with additional models incorporating sex as a predictor. In all cases the response variable was the final KDM‐derived δAge values (i.e., post‐intervention). Statistical significance was determined at *p* < 0.05. Within‐individual Spearman correlations were calculated between ΔKDM‐derived δAge and changes in metabolic and inflammatory biomarkers; full results are provided in Supplementary Figure S1 and Table S1.

## 3 Results

### 3.1 Performance of Different KDM Estimators in NHANES

**FIGURE 1:**
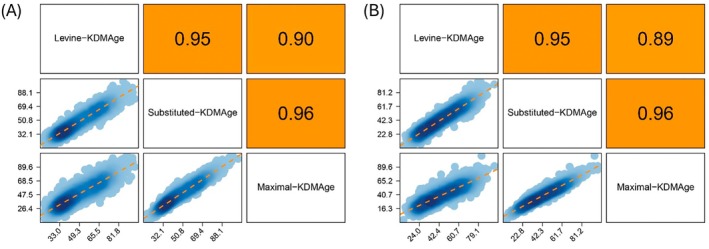
Correlations between KDMAge algorithms in NHANES III (A) and NHANES IV (B). KDM = Klemera‐Doubal Method. KDMAge = age‐relative physiological status estimated using the KDM applied to CA‐correlated biomarkers.

**FIGURE 2:**
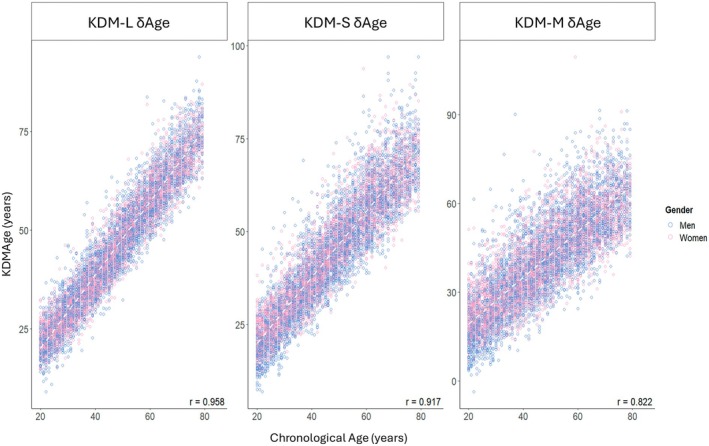
KDMAge versus Chronological Age (CA) in NHANES IV. Each panel shows results for KDMAge estimates calculated using a different set of biomarkers. NHANES III was used as the training dataset for all three algorithms. KDMAge = age‐relative physiological status estimated using the Klemera‐Doubal Method (KDM) applied to CA‐correlated biomarkers.

To validate the generalizability of our KDM‐derived δAge measures, we tested their performance in external datasets (i.e., the same final NHANES datasets used to calculate KDM values in the BioAge package). In the NHANES III training data and NHANES IV test data, all KDMAge measures demonstrated strong positive correlations with one another (Figure 1). Importantly, in NHANES IV (i.e., the test population) all KDM estimators positively correlated with CA (Figure 2); our new measures of, KDM‐S and KDM‐M, had high correlation coefficients of 0.92 and 0.82, respectively. Though we note that these were slightly lower than the original KDM‐L as used by Levine (2013), which had a correlation coefficient of 0.96 with CA.

**FIGURE 3:**
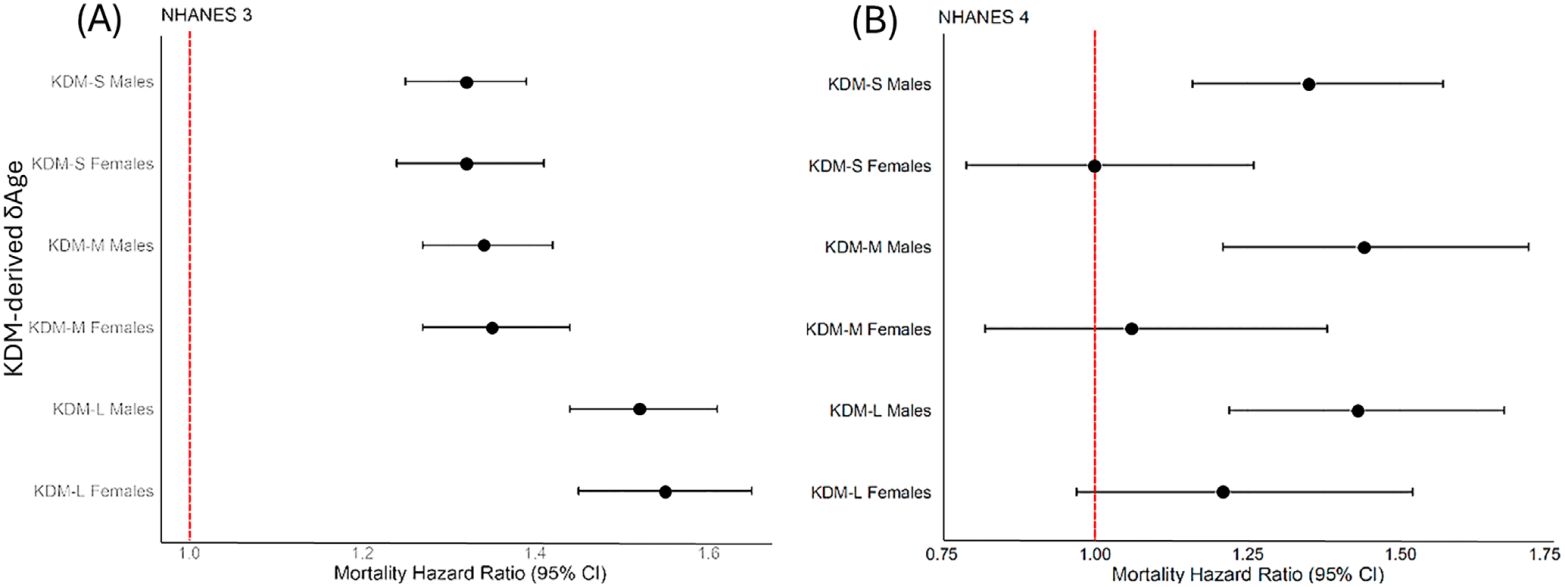
Associations between Klemera‐Doubal Method (KDM)‐derived δAge and mortality in NHANES III and IV populations. Associations of KDM‐derived δAge with mortality in the NHANES III reference population (A) and NHANES IV test population (B). KDMAge measures were calculated for non‐pregnant NHANES III participants, aged 30–75. Hazard ratios were estimated using Cox proportional hazard models. δAge measures were calculated as the difference of KDMAge from chronological age (CA). To express effect sizes in terms of a sex‐specific increase of 1 standard deviation in δAge, δAge values were standardised sex specifically within the analysis sample to have a mean of 0 and standard deviation of 1. KDM = Klemera‐Doubal Method. KDMAge = age‐relative physiological status estimated using the KDM applied to CA‐correlated biomarkers.

The associations of KDM‐derived δAge measures with mortality were examined in a sex‐specific manner in both the NHANES III reference population and the NHANES IV test population. In NHANES III, all KDM measures demonstrated a statistically significant MHR greater than 1.0, regardless of sex. Hence, KDM‐derived δAge is predictive of mortality risk in the training dataset, regardless of either the subset of biomarkers used to calculate it, or sex (Figure 3A). However, KDM‐L, which was based on Levine' (2013) original set of biomarkers, was associated with a higher mortality risk than δAge calculated using the KDM‐S and KDM‐M algorithms.

In the NHANES IV test population, MHRs varied more between sexes and KDM algorithms (Figure 3B). In NHANES IV, none of the algorithms had statistically significant MHRs for females, with KDM‐S having an MHR of 1. The KDM‐L algorithm performed as expected, producing MHRs mirroring the results seen in Kwon and Belsky's (2021) analysis using the same algorithm and population. For males in NHANES IV, all estimators for KDM were positive and statistically significant. The highest MHR in males (1.44, 95% CI: 1.21–1.71) indicated a 44% increase in morality risk associated with a 1SD increase in KDM‐M‐derived δAge. The effect in males was of similar magnitude for KDM‐L, though was slightly weaker for KDM‐S.

### 3.2 Effects of Diet on KDM‐Derived δAge in the NHL Trial

**FIGURE 4:**
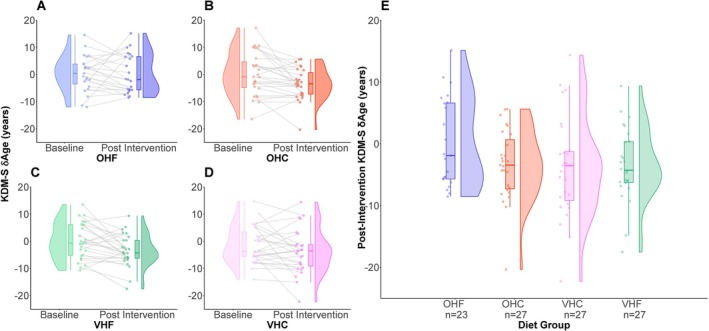
Impact of dietary interventions on KDM‐S‐derived δAge in NHL participants (A–D) show individual data points, before and after intervention as well as the density, distribution and median of the participants' KDM‐S‐derived δAge values. The box plots show the median and distribution of the δAge data within each diet group, while the half violin plots show the shape of each distribution. Each plot displays KDM‐S‐derived δAge outcomes for participants in a single dietary intervention group: OHF, OHC, VHF or VHC, from left to right and top to bottom, respectively. (E) shows post intervention KDM‐S‐derived δAge for the different dietary intervention groups. δAge represents the difference of KDMAge from CA, with δAge ≥ 0 indicating KDMAge estimates greater than CA. KDMAge was estimated using the KDM‐S algorithm. KDM‐S was calculated based on the following biomarkers: Serum glucose, serum albumin, serum total cholesterol, serum c‐reactive protein, urinary creatinine and systolic blood pressure. VHC = vegetarian, high carb. VHF = vegetarian, high fat. OHC = omnivorous high carb. OHF = omnivorous high fat. KDM = Klemera‐Doubal Method. KDMAge = age‐relative physiological status estimated using the KDM applied to CA‐correlated biomarkers. *n* indicates the number of participants in each dietary intervention group for which KDM‐S‐derived δAge was calculated.

**FIGURE 5:**
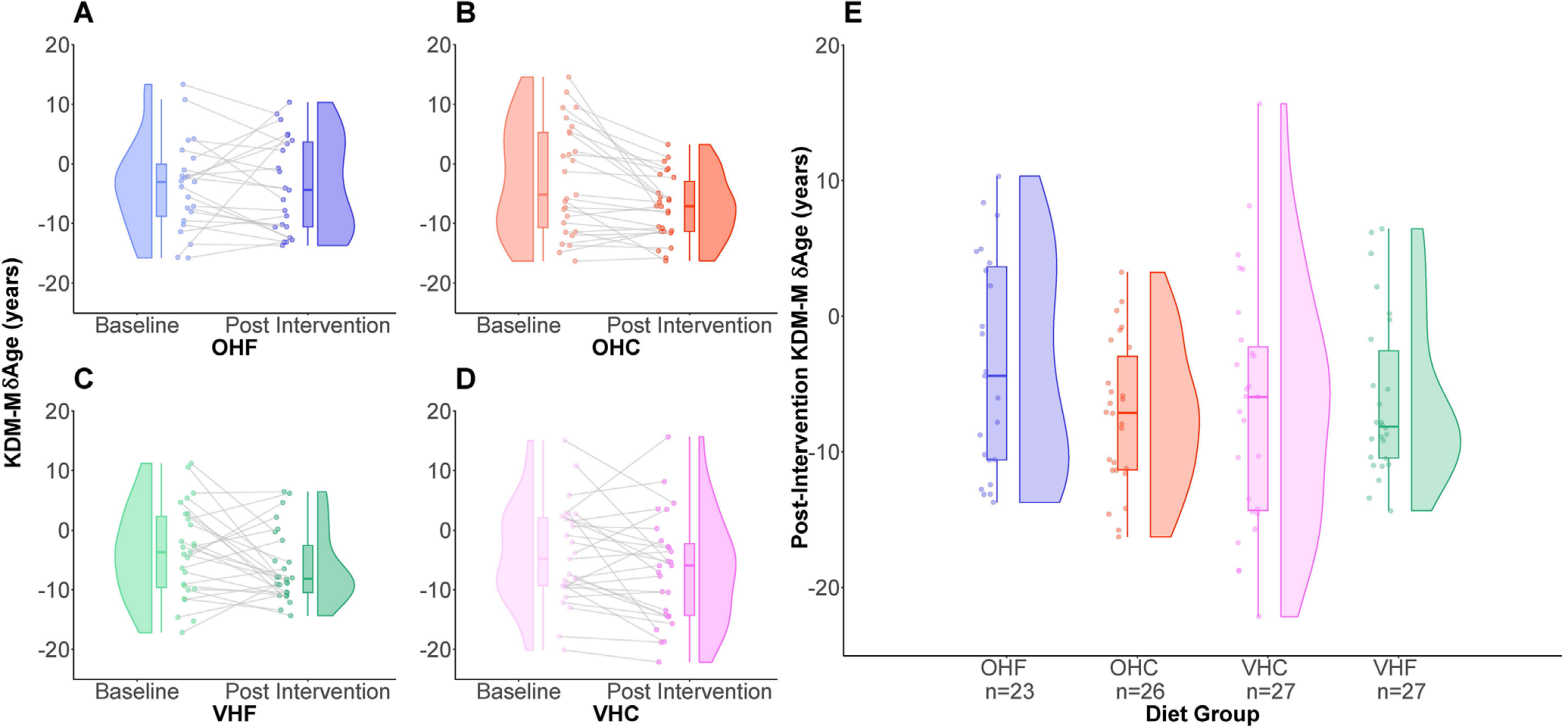
Impact of dietary interventions on KDM‐M‐derived δAge in NHL participants (A–D) show individual data points, before and after intervention as well as the density, distribution and median of the participants' KDM‐M‐derived δAge values. Each plot displays KDM‐M‐derived δAge outcomes for participants in a single dietary intervention group: OHF, OHC, VHF or VHC, from left to right and top to bottom, respectively. (E) shows post intervention KDM‐M‐derived δAge for the different dietary intervention groups. δAge represents the difference of KDMAge from CA, with δAge > 0 indicating KDMAge estimates greater than CA. KDMAge was estimated using the KDM‐M algorithm. KDM‐M was calculated based on the following biomarkers: Serum c‐reactive protein, serum albumin, serum total cholesterol, systolic blood pressure, serum HDL cholesterol, diastolic blood pressure, serum insulin, serum alkaline phosphatase, aspartate aminotransferase to alanine aminotransferase ratio, serum total bilirubin, serum triglycerides, serum glucose, urinary creatinine, waist circumference and body mass index. VHC = vegetarian, high carb. VHF = vegetarian, high fat. OHC = omnivorous high carb. OHF = omnivorous high fat. KDM = Klemera‐Doubal Method. KDMAge = age‐relative physiological status estimated using the KDM applied to CA‐correlated biomarkers. n indicates the number of participants in each dietary intervention group for which KDM‐M‐derived δAge was calculated. Note, one participant in the OHC dietary intervention group had sufficient biomarker data to calculate KDM‐S‐derived δAge but not KDM‐M‐derived δAge.

At baseline, δAge was, on average, negative, indicating that the NHL participants have a typically lower KDM‐derived δAge than the participants in NHANES, the reference population upon which the KDM algorithms are trained. On average and relative to baseline, participants on the omnivorous, high‐fat (OHF) diet showed no significant change in 𝛿Age (Figures 4A and 5A). In contrast, the average change in 𝛿Age from baseline in the other three diets was negative, indicating a shift toward lower KDM‐derived δAge values within participants on these diets (Figures 4B–D and 5B–D). Linear regression models that compared the final 𝛿Age among groups, after correction for baseline 𝛿Age indicated statistically significant differences between diet groups. Compared to the reference OHF diet group, the omnivorous high‐carb (OHC) diet group showed statistically significant reductions in both KDM‐S and KDM‐M‐derived 𝛿Age (Figures 4E and 5E; Tables 2 and 3). The high‐carb and high‐fat vegetarian (VHC and VHF, respectively) diet groups also showed reduced KDM‐S and KDM‐M‐derived 𝛿Age relative to the OHF diet group. In the case of KDM‐S‐derived 𝛿Age the difference between the VHF and OHF diet groups was statistically significant (Table 2), though that for KDM‐M‐derived 𝛿Age (for which we have one fewer data) was non‐significant (Table 3). While the VHC group had lower measures of δAge than the OHF group, on average, this difference was not significant for either KDM‐S or KDM‐M (Table 2; Table 3). Including sex as a co‐variate in the models slightly improved the model fit, as measured by the increase in adjusted *R*‐squared values from 0.314 and 0.372 to 0.322 and 0.381, respectively. However, it did not alter conclusions around the differences between the OHF group and the other diet groups (Table 2 vs. Table 4; Table 3 vs. Table 5).

**TABLE 2:** Linear regression results for final KDM‐S‐derived δAge.

	Coefficient	Standard error	*t* value	*p*
Final KDM‐S‐derived δAge				
Intercept	0.001	1.188	0.001	0.999
Baseline KDM‐S δAge	0.533	0.081	6.564	< 0.001***
Diet (OHC)	−3.513	1.616	2.173	0.032*
Diet (VHC)	−3.140	1.622	1.935	0.056^†^
Diet (VHF)	−3.515	1.617	2.174	0.032*
Regression statistics				
Multiple *R* ^2^	0.340			
Adjusted *R* ^2^	0.314			
Residual standard error	5.969			
*F*‐statistic ($F_{4,99}$)	12.770		*p*	< 0.001***
Observations	104			

*Note:* Results of multiple linear regression analysis for the KDM‐S algorithm, which predicts post‐intervention δAge based on baseline δAge and dietary interventions. The table includes the estimated coefficients, standard errors, *t*‐values and *p*‐values for each predictor variable. Regression statistics, including multiple R‐squared, adjusted R‐squared, residual standard error, *F*‐statistic and the number of observations, are also provided. Significance codes are as follows: ***p < 0.001, *p < 0.05 and ^†^ *p* < 0.1.

**TABLE 3:** Linear regression results for final KDM‐M‐derived δAge.

	Coefficient	Standard error	*t* value	*p*
Final KDM‐M‐derived δAge				
Intercept	−1.644	1.261	−1.272	0.206
Baseline KDM‐M δAge	0.552	0.072	7.689	< 0.001***
Diet (OHC)	−4.149	1.692	−2.452	0.016*
Diet (VHC)	−2.932	1.676	−1.749	0.083^†^
Diet (VHF)	−3.223	1.676	−1.923	0.057^†^
Regression statistics				
Multiple *R* ^2^	0.396			
Adjusted *R* ^2^	0.372			
Residual standard error	5.906			
*F*‐statistic ($F_{4,98}$)	16.08		*p*	< 0.001***
Observations	103			

*Note:* Results of multiple linear regression analysis for the KDM‐M algorithm, which predicts post‐intervention δAge based on baseline δAge and dietary interventions. The table includes the estimated coefficients, standard errors, *t*‐values and *p*‐values for each predictor variable. Regression statistics, including multiple R‐squared, adjusted R‐squared, residual standard error, *F*‐statistic and the number of observations, are also provided. Significance codes are as follows: ***p < 0.001, *p < 0.05 and ^†^ *p* < 0.1.

**TABLE 4:** Linear regression results for Final KDM‐S‐derived δAge, including sex.

	Coefficient	Standard error	*t* value	*p*
Final KDM‐S‐derived δAge				
Intercept	−0.617	1.251	−0.494	0.623
Baseline KDM‐S δAge	0.535	0.081	6.624	< 0.001***
Diet (OHC)	−3.487	1.606	−2.171	0.032*
Diet (VHC)	−3.045	1.614	−1.887	0.062^†^
Diet (VHF)	−3.489	1.607	−2.172	0.032*
Sex (Male)	1.775	1.184	1.499	0.137
Regression statistics				
Multiple *R* ^2^	0.355			
Adjusted *R* ^2^	0.322			
Residual standard error	5.661			
*F*‐statistic ($F_{5,98}$)	10.800		*p*	< 0.001***
Observations	104			

*Note:* This table presents the results of the multiple linear regression analysis predicting final KDM‐S‐derived δAge based on baseline KDM‐S‐derived δAge, dietary interventions, and sex. The reference diet is the omnivorous high‐fat (OHF) diet. The table includes the estimated coefficients, standard errors, *t*‐values and *p*‐values for each predictor variable. Regression statistics, including multiple *R*‐squared, adjusted *R*‐squared, residual standard error, *F*‐statistic and the number of observations, are also provided. Significance codes are as follows: ***p < 0.001, *p < 0.05 and ^†^ *p* < 0.1.

**TABLE 5:** Linear regression results for final KDM‐M‐derived δAge, including sex.

	Coefficient	Standard error	*t* value	*p*
Final KDM‐M‐derived δAge				
Intercept	−2.271	1.330	−1.707	0.091
Baseline KDM‐M δAge	0.543	0.072	7.593	< 0.001***
Diet (OHC)	−4.054	1.680	−2.413	0.018*
Diet (VHC)	−2.822	1.664	−1.696	0.093
Diet (VHF)	−3.216	1.663	−2.934	0.056
Sex (Male)	1.838	1.243	1.479	0.142
Regression statistics				
Multiple *R* ^2^	0.411			
Adjusted *R* ^2^	0.381			
Residual standard error	5.860			
*F*‐statistic ($F_{5,97}$)	13.540		*p*	< 0.001***
Observations	103			

*Note:* This table presents the results of the linear regression analysis predicting final KDM‐M‐derived δAge based on baseline KDM‐M‐derived δAge, dietary interventions and sex. The reference diet is the omnivorous high‐fat (OHF) diet. The table includes the estimated coefficients, standard errors, *t*‐values and *p*‐values for each predictor variable. Regression statistics, including multiple *R*‐squared, adjusted *R*‐squared, residual standard error, *F*‐statistic and the number of observations, are also provided. Significance codes are as follows: ***p < 0.001, and *p < 0.05.

## 4 Discussion

This study aimed to assess whether short‐term dietary manipulation could influence KDM‐derived δAge, a composite biomarker that integrates multi‐system health and is often used as a proxy for biological age in large epidemiological cohorts (Parker et al. 2020; Murabito et al. 2018). While δAge has been associated with longer‐term morbidity and mortality outcomes (Levine 2013; Murabito et al. 2018; Belsky et al. 2015; Liu et al. 2018; Talifu et al. 2025; Nie et al. 2022), its responsiveness in the context of short‐term interventions remains uncertain (Pham et al. 2024). We observed that in an older population, KDM‐derived δAge responds to dietary intervention over just 4 weeks. However, whether these changes represent meaningful shifts in ageing biology or transient physiological fluctuations remains an open question.

KDM‐derived δAge integrates biomarkers influenced by metabolic, cardiovascular and inflammatory states (Yang et al. 2026; Jiang et al. 2024); systems that are highly sensitive to short‐term dietary changes (Fewkes et al. 2022; Pointke et al. 2024; Giugliano et al. 2006). As such, observed improvements in δAge could reflect acute physiological responses rather than a reversal of age‐related dysfunction. For example, a meal with specific fatty acid profiles or altered salt content can influence biomarker levels transiently without fundamentally altering disease risk (He and MacGregor 2009). Such mutable causes align with the known adaptive nature of physiological regulation, whereby many of the biomarkers contributing to KDMAge estimates are homeostatically controlled and can shift rapidly in response to factors such as diet, physical activity, stress or medication. These changes serve an adaptive function, and though they may be transiently reflected in composite indices like δAge, they do not necessarily indicate lasting changes in the underlying pathologies of ageing. Therefore, while δAge decreased in response to specific dietary patterns, it is difficult to distinguish whether the observed changes reflect the immediate impact of nutritional inputs on integrated physiological systems or a systematic improvement in ageing‐related health. This study was not, however, designed to disentangle these causes, nor establish whether δAge changes persist after the intervention or predict long‐term outcomes. It does, nonetheless, indicate that any interpretation of δAge as a proxy for physiological resilience must be made cautiously, cognisant of the fact that biomarker responses could reflect both acute physiological responses to recent dietary exposures and longer‐term changes to the state of the biological system more closely aligned with biological ageing.

In this regard, the consistency and direction of any transient and longer‐term effects is interesting. The NHL diets that were associated with reductions in δAge have been associated with reduced chronic disease risk in numerous studies. For example, reductions in δAge were observed in response to semi‐vegetarian and high‐carbohydrate diets, patterns that have been linked in other contexts to improved metabolic health and reduced risk of chronic disease (Petermann‐Rocha et al. 2021; Appleby and Key 2016; Kahleova et al. 2018). The OHC diet, in particular, led to significant δAge reductions relative to the reference OHF diet, consistent with prior evidence that diets rich in complex carbohydrates can extend lifespan and improve metabolic health across species (Kitada et al. 2019; Le Couteur et al. 2016; Levine et al. 2014; Wali et al. 2021, 2024). This raises interesting questions about how short‐term, transient responses to favourable or deleterious dietary exposures may relate to sustained changes that predict mortality risk, which are more intuitively associated with ageing. It also suggests that δAge may serve as a responsive indicator of dietary impact on age‐relative systemic health, even if not yet a definitive marker of actual biological ageing (Belsky et al. 2017; Belsky 2017), particularly when interpreted alongside broader patterns of biomarker and health change.

The carbohydrates in the NHL diets were tightly controlled and primarily comprised complex carbohydrates, derived from whole, minimally processed sources. Therefore, our findings should not be extrapolated to diets high in refined or simple carbohydrates, which may elicit different metabolic effects (Wali et al. 2021, 2024). Similar trends in δAge improvement were observed in the VHC and VHF groups, although not all results reached statistical significance. While some comparisons approached (but did not reach) conventional thresholds (e.g., *p* = 0.055), the consistent direction of effects across groups supports the hypothesis that dietary composition influences age‐related physiological health status, suggesting potential biological relevance that warrants further investigation in larger samples.

These outcomes also align with studies suggesting that plant‐based proteins are associated with lower levels of biomarkers linked to inflammation and oxidative stress, factors relevant to ageing biology (Wang, Li, et al. 2023; Dwaraka et al. 2024; Hruby and Jacques 2019; Eichelmann et al. 2016; Elliott et al. 2022). However, this study was not designed to assess these mechanisms directly. While protein source may influence δAge, the balance of dietary fat and fibre may play an important role in influencing physiological ageing profiles. Diets higher in fibre are typically lower in fat and energy density (Wali et al. 2024; Belsky et al. 2017) and such characteristics may enhance metabolic health by improving insulin sensitivity, reducing inflammation and supporting healthier lipid profiles, factors that KDM‐derived δAge has been shown to capture (Zhu et al. 2025). This suggests that the benefits of plant‐forward diets may derive not only from specific ingredients, but from broader shifts in macronutrient quality and composition.

This interpretation is reinforced by other findings from the NHL trial. Ribiero et al. reported that all NHL intervention diets differed substantially from participants' habitual dietary patterns, with the exception of the OHF diet, which most closely resembled participants' habitual dietary patterns and showed no observed change in δAge from baseline. Participants' pre‐intervention diets reflected typical Australian dietary profiles, characterised by high levels of processed foods, refined sugars and saturated fats (Machado et al. 2019). In contrast, the intervention diets emphasised whole foods, increased fibre and reduced fat and animal‐based protein. The diets that led to δAge reductions (OHC, VHC and VHF) also showed the greatest divergence from baseline in terms of fat and fibre content (toward lower fat and higher fibre). These changes were accompanied by improvements in body composition and cardiometabolic biomarkers, particularly in the pro‐vegetarian groups, suggesting systemic physiological benefit (Ribeiro et al. 2024).

Moreover, our findings align with broader literature linking high‐fibre, plant‐forward diets to lower inflammation, improved metabolic function and reduced age‐related disease risk (Slavin 2013; Anderson et al. 2009; McKeown et al. 2022; Koloverou et al. 2016). For instance, a recent meta‐analysis of controlled dietary fibre intervention trials reported benefits across a range of health indicators, including reductions in all‐cause mortality (Reynolds et al. 2022). Similarly, cohort studies have shown associations between increased fibre intake and reduced risks of chronic diseases such as cardiovascular disease and type‐2 diabetes (Chen, Zhang, Hu, et al. 2023; Fatima et al. 2023; Reynolds et al. 2019). Zhu et al. also reported that higher intake of plant protein, fibre and high‐quality carbohydrates was associated with improved biomarker‐based composite ageing metrics.

Western dietary patterns, including the standard Australian diet, are typically low in fruits, vegetables and whole grains (key sources of fibre, complex carbohydrates and other key anti‐inflammatory nutrients) and high in calorie‐dense, ultra‐processed foods and pro‐inflammatory components. In addition to their higher fibre content, the OHC, VHC and VHF diets included more servings of fruits, vegetables and legumes, compared to the OHF diet. Pro‐inflammatory diets have been shown to elevate composite ageing estimates like KDM and PhenoAge (Xie et al. 2023), supporting possible anti‐inflammatory systemic shifts of the OHC, VHC and VHF diets. These observations are externally coherent with our findings, suggesting that the dietary interventions that lowered δAge also align with health‐promoting nutritional patterns seen in broader epidemiological research (Fabiani et al. 2019; Koelman et al. 2022).

Importantly, the interpretation of δAge as a signal of systemic ageing is informed by the characteristics of the biomarkers used in the KDM‐S and KDM‐M algorithms. Several included markers are known to be responsive to dietary changes within days to weeks, reflecting acute shifts in metabolic and inflammatory status. However, other biomarkers within these algorithms, including albumin, alkaline phosphatase, creatinine and waist circumference, tend to change more slowly and are less sensitive to short‐term fluctuations (Nakamura and Miyao 2007; Crimmins et al. 2008). The inclusion of both rapidly responsive and more temporally stable indicators reduces the likelihood that δAge merely reflects transient physiological noise (Kulminski et al. 2008). Instead, the observed changes likely reflect coordinated shifts across multiple physiological systems. The consistency across both the KDM‐S and KDM‐M variants, despite their different biomarker compositions, strengthens this interpretation that δAge captured meaningful changes in systemic health rather than artefacts of isolated or short‐term biomarker variability. The KDM‐M algorithm, which included additional markers such as insulin and HDL, yielded comparable results to the KDM‐S estimates, suggesting the observed changes reflect a broader shift in systemic integrity rather than narrow or isolated effects (Belsky et al. 2017). For example, although Ribeiro et al. reported an effect of dietary protein on DBP across NHL participants, our findings remained robust regardless of DBP inclusion. This supports prior evidence that the KDM algorithm captures patterns of system‐wide physiological dysregulation relevant to ageing biology (Yokoyama et al. 2014; Tian et al. 2025).

While some may view the reversibility of δAge as evidence against its biological relevance, recent studies suggest that plasticity in biological age estimates may reflect dynamic health responsiveness. For example, Komaki et al. (2022) demonstrated that epigenetic clocks can exhibit short‐term fluctuations in response to physiological changes, and Poganik et al. (2023) showed that such clocks increase with acute stress and reverse with recovery, supporting their role as integrated barometers of systemic state. Moqri et al. (2023) further proposed that valid ageing biomarkers should track chronological age, predict future health outcomes and respond to intervention. While no single study establishes all three criteria simultaneously, the convergence of evidence across cohort and intervention data reinforces the relevance of δAge as a dynamic indicator of systemic health, even if the present trial cannot determine whether short‐term dietary responses translate into long‐term outcome differences.

These results also hold relevance given the validated predictive power of KDM estimates in large epidemiological datasets such as NHANES. The KDM algorithm was originally applied by Levine (Levine 2013) using NHANES data and has demonstrated predictive validity for age‐related outcomes across multiple datasets (Wei et al. 2022; Parker et al. 2020; Levine 2013; Belsky et al. 2020; Chen, Zhang, Yu, et al. 2023). When applied to NHANES III and IV, the modified variants were highly correlated with the original KDM‐L and demonstrated comparable predictive performance, highlighting the algorithm's adaptability across biomarker sets (Parker et al. 2020; Kwon and Belsky 2021). Consistent mortality hazard ratios across versions (Levine 2013; Kwon and Belsky 2021; Jiang et al. 2024; Earls et al. 2019) reinforce the interpretation that δAge captures meaningful variation in systemic physiological integrity and remains relevant to age‐related health risk at the population level (Belsky et al. 2015). Its established predictive validity across diverse cohorts supports δAge as a proxy for physiological health, even if the specific mechanisms and timescales underlying its shifts remain unresolved (Nie et al. 2022).

Importantly, predictive validity was strongest in males in NHANES IV, suggesting potential sex‐specific differences in the utility of δAge as a predictor of long‐term health outcomes (Wei et al. 2022; Machado et al. 2024; Tzemah‐Shahar et al. 2024). This highlights the importance of considering sex‐stratified or sex‐corrected analyses (Hägg and Jylhävä 2021; Phyo et al. 2024; Iannuzzi et al. 2023; Hu et al. 2025; Moqri et al. 2024) when interpreting intervention effects in the NHL study and strengthens the significance of any observed δAge change in male participants, given the clearer association between δAge and survival in this subgroup. Together, these findings provide essential context for interpreting δAge within this trial as a validated and scalable proxy with demonstrated relevance to morbidity and mortality risk.

### 4.1 Strengths and Limitations

A key strength of this study lies in the high level of dietary control during the intervention. Nearly all food consumed was provided to participants, and intake was measured using weighed food records, enhancing internal validity and enabling confident attribution of observed δAge shifts to the intervention itself. Strict inclusion criteria further minimised external variability, supporting the reliability of the findings. The use of multiple KDM variants also strengthens the robustness of the findings.

While δAge is correlated with age‐related morbidity and mortality in large cohorts, its interpretation in short‐duration trials requires caution. As discussed above, changes in δAge may reflect group‐level physiological responsiveness rather than irreversible biological ageing or improvements in long‐term health outcomes. Put simply, δAge may be a marker of the diet, rather than reflecting the effects of the diet on long‐term outcomes (Nowlin et al. 2012).

Despite the strengths of the intervention, the high internal validity comes with trade‐offs in generalisability. The relatively small sample size, particularly for the modified KDM‐M algorithm (due to missing data), may have limited statistical power and contributed to non‐significant findings in some cases. Additionally, the NHL participants represent a relatively healthy sub‐population at baseline, with negative δAge values on average, suggesting more resilient physiological states. This limits extrapolation to broader populations. Furthermore, while the KDM framework has demonstrated predictive validity across diverse independent cohorts, including the UK Biobank (Mak et al. 2023; Chan et al. 2021) and the China Kadoorie Biobank (Chen, Zhang, Yu, et al. 2023), the specific biomarker combinations used in KDM‐S and KDM‐M have not been independently validated outside NHANES. However, their application to the Australian NHL cohort represents a degree of external testing, and the consistency of results across both algorithm variants provides reassurance that findings are not driven by idiosyncratic biomarker behaviour or artefacts of the NHANES measurement environment. Future research should explore whether these findings extend to more diverse cohorts and whether short‐term δAge changes are sustained or predictive of long‐term outcomes (Zhu et al. 2024). Integrating complementary ageing metrics, such as epigenetic clocks based on DNA methylation (Fatima et al. 2023; Reynolds et al. 2019), may also offer deeper insights into molecular ageing processes and their responsiveness to lifestyle interventions.

## 5 Conclusions

The present findings show that δAge is modifiable in response to short‐term dietary intervention, with the most pronounced improvements seen in diets rich in complex carbohydrates and plant‐based components. While these effects may partly reflect transient physiological states, they also align with long‐term dietary patterns known to reduce disease risk and promote systemic health. As such, δAge may capture acute, systemic responses to beneficial dietary changes that, if sustained, could accumulate into more durable improvements in physiological function, though whether such accumulation occurs remains to be established in longer term trials. More broadly, disentangling short‐term responsiveness from true ageing‐related change remains essential, and future research must clarify the predictive value of δAge in intervention settings.

## Author Contributions

C.J.A. co‐designed the analysis, conducted the analysis, interpreted the results and prepared the manuscript. A.G., R.V.R., S.J.S., D.L.C. and D.R. contributed to the randomised controlled trial protocol. A.G. and R.V.R. collected biological samples, and J.T. quantified biomarkers in the laboratory. S.J.S., D.R. and A.M.S. contributed to result interpretation and manuscript revision.

## Funding

C.J.A. was supported by the University of Sydney, Faculty of Science Research Stipend Scholarship (SC3852). A.M.S. received support from the University of Sydney Horizon Program and the Australian Research Council Future Fellowship scheme. The NHL study was supported by Emeritus Professor George Palmer [philanthropic donation]. R.V.R. was supported by a Charles Perkins Centre Early Career Fellowship from Ms. Jennie Mackenzie. The funders played no role in the design, execution, analysis, and interpretation of data, or writing of the NHL study.

## Conflicts of Interest

The authors declare no conflicts of interest.

## Supporting information


**Figure S1:** Correlogram of within‐individual Spearman correlations between ΔKDM‐derived δAge and changes in CRP, glucose, insulin, HOMA‐IR, total cholesterol, HDL, LDL, triglycerides and waist circumference, among NHL participants. ΔKDM‐derived δAge = difference between pre‐ and post‐intervention KDM‐derived δAge values.
**Table S1:** Within‐individual Spearman correlations between ΔKDM‐derived δAge and changes in metabolic/inflammatory biomarkers, among NHL participants. Biomarker changes measured as difference between post‐intervention and pre‐intervention values. ΔKDM‐derived δAge = difference between pre‐ and post‐intervention KDM‐derived δAge values.

## Data Availability

The data that support the findings of this study are available on request from the corresponding author. The data are not publicly available due to privacy or ethical restrictions. Publicly available data from the National Health and Nutrition Examination Survey (NHANES) were also used in this study and can be accessed directly from the Centers for Disease Control and Prevention [https://www.cdc.gov/nchs/nhanes/].
