# Supplementary material for: Short‐Term Dietary Intervention Alters Physiological Profiles Relevant to Ageing
Source: Aging Cell. 2026 Apr 27;25(5):e70507. doi: 10.1111/acel.70507 (PMC13112187; doi:10.1111/acel.70507)

**Supplementary Materials**


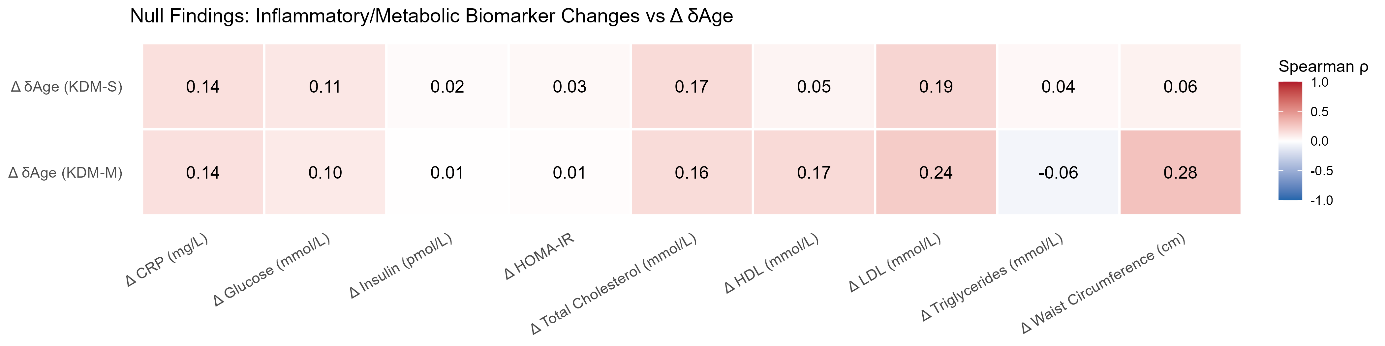


**Supplementary Figure 1.** Correlogram of within-individual Spearman correlations between ΔKDM-derived δAge and changes in CRP, glucose, insulin, HOMA-IR, total cholesterol, HDL, LDL, triglycerides, and waist circumference, among NHL participants. ΔKDM-derived δAge = difference between pre- and post-intervention KDM-derived δAge values.

**Supplementary Table 1.** Within-individual Spearman correlations between ΔKDM-derived δAge and changes in metabolic/inflammatory biomarkers, among NHL participants. Biomarker changes measured as difference between post-intervention and pre-intervention values. ΔKDM-derived δAge = difference between pre- and post-intervention KDM-derived δAge values.


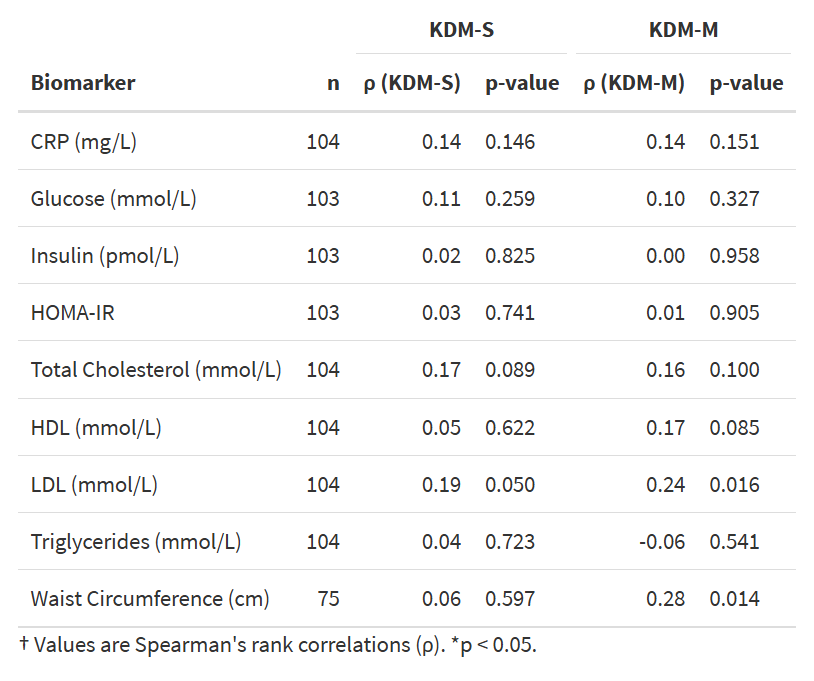

Supplement: Supplementary file 1 — Figure S1: Correlogram of within‐individual Spearman correlations between ΔKDM‐derived δAge and changes in CRP, glucose, insulin, HOMA‐IR, total cholesterol, HDL, LDL, triglycerides and waist circumference, among NHL participants. ΔKDM‐derived δAge = difference between pre‐ and post‐intervention KDM‐derived δAge values. Table S1: Within‐individual Spearman correlations between ΔKDM‐derived δAge and changes in metabolic/inflammatory biomarkers, among NHL participants. Biomarker changes measured as difference between post‐intervention and pre‐intervention values. ΔKDM‐derived δAge = difference between pre‐ and post‐intervention KDM‐derived δAge values. [file ACEL-25-e70507-s001.docx]
